# Technologies for Monoclonal Antibody Discovery and Development

**DOI:** 10.3390/ijms262110470

**Published:** 2025-10-28

**Authors:** Kyung Ho Han, Yi-Chuan Li, Rabia Parveen, Srimathi Venkataraman, Chih-Wei Lin

**Affiliations:** 1 Department of Biological Sciences and Biotechnology, Hannam University, Daejeon 34054, Republic of Korea; 2 Department of Biological Science and Technology, China Medical University, Taichung 406040, Taiwan; 3 Cancer Biology and Precision Therapeutics Center, China Medical University, Taichung 406040, Taiwan; 4 International Master Program of Biomedical Sciences, China Medical University, Taichung 406040, Taiwan; 5 Graduate Institute of Biological Science and Technology, China Medical University, Taichung 406040, Taiwan; 6 Institute of Biochemistry and Molecular Biology, China Medical University, Taichung 406040, Taiwan

**Keywords:** antibody, hybridoma, phage display, antibody engineering, de novo synthesis

## Abstract

Monoclonal antibodies (mAbs) represent one of the most successful classes of biopharmaceuticals, with more than 100 approved for treating oncological, immunological, and infectious diseases. Antibody discovery and development have been driven by diverse methodologies. Classical strategies such as mouse hybridoma technology, phage display, transgenic mouse models, and single B cell isolation have enabled the generation of high-affinity therapeutic antibodies. Beyond binding affinity, recent innovations in combinatorial antibody libraries have facilitated the selection of functional antibodies within cellular environments, revealing their ability to act as agonists or antagonists and influence signal transduction pathways. These insights expand therapeutic applications by enabling modulation of complex cellular responses. Recent breakthroughs in artificial intelligence, involving antibody generation supported by rapidly growing antibody sequence and structure databases, are transforming computational protein design. This review highlights five major approaches (hybridoma technology, phage display, transgenic mouse models, and single B cell isolation, de novo antibody design) for antibody discovery and development. These approaches offer innovative strategies designed to accelerate the discovery process and enhance therapeutic outcomes for human diseases.

## 1. Introduction

**Figure 1:**
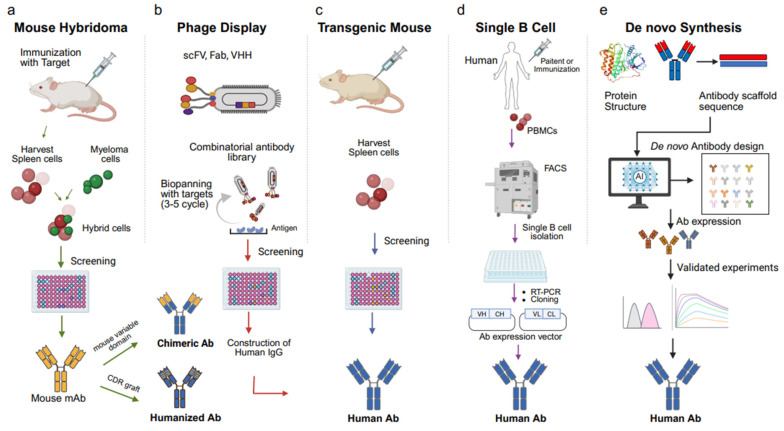
Overview of five key approaches for therapeutic antibody development: (**a**) mouse hybridoma, (**b**) phage display, (**c**) transgenic mouse, (**d**) single B cell isolation, and (**e**) de novo design. Each method enables monoclonal antibody generation through distinct immunization, screening, or computational strategies. (**a**) Mouse hybridoma technology: Mice are immunized with the target antigen, and spleen B cells are fused with immortal myeloma cells to generate hybridomas. Hybridoma clones are screened for specific antibody production, followed by antibody humanization through complementarity-determining region (CDR) grafting. (**b**) Phage display: A combinatorial antibody library was generated in single-chain variable fragment (scFv), fragment antigen-binding (Fab), or single-domain antibody (VHH) formats. Repeated rounds of biopanning against immobilized antigens enrich high-affinity binders for screening and IgG reconstruction. (**c**) Transgenic mouse platforms: Genetically engineered mice expressing human immunoglobulin genes are immunized with target antigens. Human antibody-producing B cells are isolated from the spleen and screened to obtain fully human antibodies. (**d**) Single B cell isolation: Peripheral blood mononuclear cells (PBMCs) are collected from immunized or patient human donors. Antigen-specific B cells are isolated by fluorescence-activated cell sorting (FACS), and antibody genes are amplified via reverse transcription polymerase chain reaction (RT-PCR) and cloned into expression vectors to obtain fully human antibodies. (**e**) De novo antibody design: Using artificial intelligence (AI) and structure-based modeling, antibody sequences are generated computationally from scaffold structures. Predicted antibodies are expressed and experimentally validated for affinity, stability, and function.

Antibodies are essential components of the adaptive immune system produced by B cells to specifically recognize and neutralize diverse antigens such as viruses, pathogens, and malignant cells. Since the pioneering work of Köhler and Milstein in developing hybridoma technology [1], monoclonal antibodies (mAbs) have become indispensable tools in both basic research and clinical medicine. To date, more than 100 therapeutic antibodies have been approved for clinical use [2], providing effective treatment options for autoimmune disorders, metabolic diseases, infectious diseases, and a wide range of cancers [2,3,4,5]. The development of therapeutic antibodies has traditionally relied on several strategies, including animal immunization, combinatorial antibody libraries, B cell cloning, and the use of transgenic mice [6,7,8]. These methods have enabled the discovery of high-affinity antibodies with desirable therapeutic properties. More recently, advances in computational biology and artificial intelligence (AI) have introduced powerful new approaches to antibody discovery [9,10,11,12]. These tools such as AlphaFold3 for structural prediction, molecular docking, and de novo design are increasingly combined with generative diffusion models to create antibodies with improved specificity, affinity, and efficacy [13]. In this review, we summarize the five major methodologies for antibody generation, these approaches illustrated in Figure 1 are expanding the landscape of therapeutic antibody discovery, offering innovative strategies to accelerate development and improve the treatment of human diseases.

## 2. Hybridoma Technology

For many years, antibodies have been an important tool in biomedical research, with potential applications in a variety of fields. The high specificity and selective binding of antibodies have expanded their application to flow cytometry, magnetic cell sorting, immunoassays, therapeutic methods, and more. In 1975, hybridoma-based technology was used to produce monoclonal antibodies with minimal inter-batch variability, allowing for continuous and unlimited production [6]. Hybridoma technology, first invented by Kohler and Milstein in 1975 as a method for obtaining monoclonal antibodies, involves fusing B cells produced in the spleen of immunized animals (such as mice) with immortalized myeloma cells [14,15]. Hybridoma technology is the original, most basic, and most successful method for isolating monoclonal antibodies. This technology is compelling and has been used to discover thousands of antibodies for various applications. The fundamental practical advantage of hybridoma technology is that once a hybridoma clone is established, the production of monoclonal antibodies becomes straightforward and efficient [16]. This technique remains extensively employed in the development of antibody therapeutics and diagnostic reagents, its core value lying in preserving the sequence integrity and biological activity of antibodies derived from the natural recombination process within B cells. The first therapeutic mAb, muromonab-CD3 (Orthoclone OKT3), was approved by the US FDA in 1986 and comprises a murine mAb against T cell-expressed CD3 that functions as an immunosuppressant for the treatment of acute transplant rejection [17]. To circumvent problems of diminished immunogenic potential and efficacy, while making possible the therapeutic use of antibodies for an extended duration, researchers developed techniques to transform rodent antibodies into structures more similar to human antibodies, without loss of binding properties. Then, the first mAb with an oncologic indication, rituximab, a chimeric anti-CD20 IgG1, was approved for the treatment of non-Hodgkin’s lymphoma in 1997 by the US FDA [18]. Recent innovations, such as electrofusion of antibody-secreting cells (ASCs) enriched via fluorescence-activated cell sorting (FACS), have significantly improved fusion efficiency and functional hybridoma yield. By selecting ASC subsets with high transmembrane activator and CAML interactor (TACI) and CD138 expression, researchers achieved more than 60% antigen-specific mAb production with nanomolar affinity. These refinements overcome prior limitations of random pairing and low PEG-mediated fusion, positioning hybridoma as a revitalized, high-yield platform for therapeutic and diagnostic antibody development [19].

In addition to mouse hybridomas, rabbit monoclonal antibodies can also be generated from hybridoma technology. Rabbit antibody shows superior affinity, broader epitope recognition including cryptic or conformational sites and greater sensitivity for therapeutic and diagnostic use [20]. Rabbit monoclonal antibodies surpass their murine counterparts in several key immunological dimensions: (1) Superior Affinity—Enhanced binding kinetics enable more precise antigen detection and targeting. (2) Expanded Epitope Recognition—Capable of identifying epitopes that are structurally complex or poorly immunogenic in mice. (3) Sensitivity to Post-Translational Modifications—Ideal for detecting subtle biochemical variations in disease states. Rabbit monoclonal antibodies (RabMAbs) offer high specificity and affinity, often recognizing epitopes inaccessible to murine antibodies, making them valuable for antibody-drug conjugates (ADCs) and checkpoint inhibitors. The FDA approval of brolucizumab, a rabbit-derived ScFv targeting VEGF-A, marked the first therapeutic RabMAb. Brolucizumab is a single-chain variable fragment (ScFv) mAb that potently inhibits VEGF. Due to their small size, ScFvs can be delivered at higher concentrations compared to other therapeutic mAb formats and can penetrate tissue more effectively to exert their therapeutic effects. A key difference that sets brolucizumab apart from other mAbs, however, is that it is a humanized ScFv derived from rabbits, making this mAb the first of its kind in the market. In contrast, most other therapeutic mAbs are derived from mice [21,22,23]. Other examples include Zilovertamab vedotin, an ADC against receptor tyrosine kinase-like orphan receptor 1 (ROR1) [24,25], and OR502, targeting leukocyte immunoglobulin-like receptor B2 (LILRB2) to enhance antitumor immunity [26,27].

## 3. Phage Display

Phage display is the first technique for in vitro antibody screening and is still the most widely used method. The technology was first described in 1985 by George P. Smith, who demonstrated that filamentous bacteriophages could present foreign peptides on their surface via DNA insertion into coat protein genes, this technology has been extensively developed. Major advances came from Winter and McCafferty at the Laboratory of Molecular Biology (Cambridge, UK), Lerner and Barbas at The Scripps Research Institute (La Jolla, USA), and Breitling and Dübel at the German Cancer Research Center (Heidelberg, Germany), who pioneered the construction of combinatorial antibody libraries in filamentous phages [28,29,30,31,32]. These milestones firmly established phage display as a robust platform for therapeutic antibody engineering, leading to the development of fully human antibodies [33,34,35,36,37,38,39,40,41,42]. Combinatorial antibody libraries are constructed by amplifying VH and VL regions from B cells, immunized donors, or patients using PCR, followed by cloning into phagemid vectors fused to phage coat proteins such as pIII. This creates a direct genotype–phenotype linkage, enabling the display of antibody fragments on the phage surface. Libraries can exceed 10^11^ variants, providing extensive diversity. Iterative rounds of binding, washing, and elution against immobilized antigens enrich antigen-specific phages, after which binders are screened by ELISA, sequenced, and reformatted as scFv, Fab, or IgG molecules for testing. Importantly, phage display bypasses immune tolerance, allowing the generation of antibodies against self-antigens such as TNF-α. The approval of Adalimumab (Humira), the first phage display-derived fully human monoclonal antibody, illustrates its clinical significance [43]. There are phage display-derived human antibodies approved by the US FDA for the treatment of human disease, demonstrating the reliability of this technique as a platform for antibody discovery [33,44,45].

**Figure 2:**
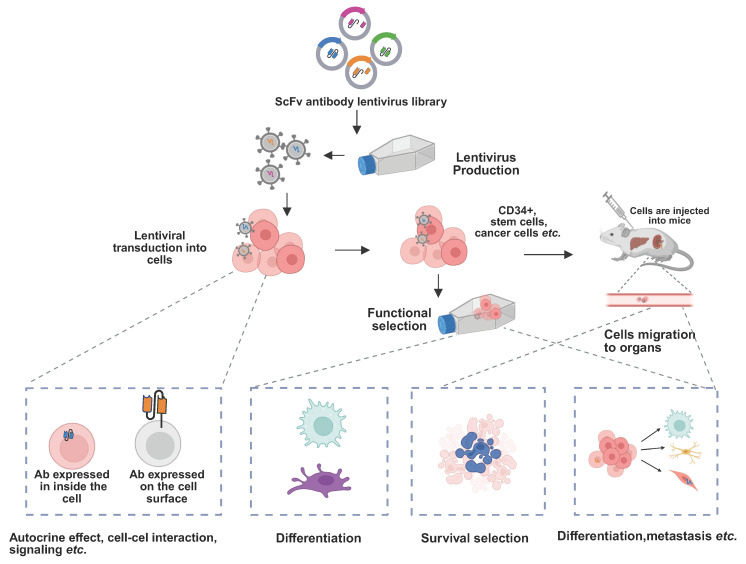
Schematic of functional antibody discovery modulating cell fate. Lentiviral delivery of antibody libraries enables intracellular and surface expression of antibodies. Functional selection in cells and in vivo identifies antibodies that modulate signaling, differentiation, and cell fate. Functional antibodies can induce changes in cell fate, with the ability to be secreted, expressed in the cytoplasm, anchored on the plasma membrane. Through the use of antibody libraries, functional antibodies can be isolated in the cellular environment and modify the function of cell surface signaling components. These antibodies can also act in an autocrine or paracrine manner to influence cell survival, proliferation, and lineage differentiation, providing a powerful platform for discovering antibodies that regulate cellular homeostasis and fate determination.

Beyond conventional binder selection, phage display has progressed to enable direct functional antibody discovery as shown as Figure 2 [46,47,48,49,50,51,52,53,54,55,56,57,58]. The identification of functional antibodies has become a central focus of research. In vivo and phenotypic screening approaches have further broadened phage display applications. Migration-based selection within whole organisms has revealed antibodies that regulate receptor pleiotropy and cell differentiation. These strategies enabled the identification of antibodies that drive stem cells into macrophage, microglia, brown adipocyte, or beta-like cell fates [52]. Morphology-based screening has even yielded antibodies that protect against virus-induced cell death [59]. In oncology, functional phage display has identified agonist antibodies activating non-canonical pathways and an anti-apoptotic intrabody against PKM2, revealing mechanisms of tumor survival [60,61,62]. These advances illustrate how phage display has evolved into a versatile discovery engine for antibodies with therapeutic functions beyond binding alone. In addition, antibodies obtained from hybridoma or phage display often undergo further engineering to improve binding. By introducing mutations into VH/VL regions and reselecting improved clones, phage display mimics natural affinity maturation that occurs in germinal centers after V(D)J recombination. This process enriches variants with higher antigen affinity, sometimes reaching low-picomolar binding strengths, and is critical for optimizing antibodies for therapeutic use [63,64].

## 4. Transgenic Mouse Technology

Antibodies derived from wild-type mice often require extensive downstream engineering to eliminate immunogenic murine sequences and to ensure proper interaction with human Fc receptors. While wild-type mice are widely available and straightforward to use, they are suboptimal for therapeutic antibody discovery. To address the challenge of immunogenicity, transgenic mouse models engineered to generate fully human antibodies. However, early generations of these models lacked murine constant regions, leading to defects in B-cell development and impaired antibody maturation within the mouse. The principle of transgenic mouse technology is to introduce human immunoglobulin (Ig) genes into the murine genome through genetic engineering, enabling mice to produce fully human antibodies. This is typically achieved by homologous recombination in embryonic stem (ES) cells or by microinjecting recombinant human antibody gene fragments into fertilized eggs, followed by embryo transfer and breeding to establish stable transgenic lines. The integrated human Ig genes cooperate with the murine immune system, ensuring normal B-cell development, somatic hypermutation, and affinity maturation. Compared with wild-type mice, these transgenic mice generate human antibodies with higher clinical relevance, reduced immunogenicity, and preserved immune surveillance. The first milestone was reported in 1989, when Brüggemann et al. [65] constructed a human heavy chain gene cassette containing two VH segments, diversity (D) elements, the JH cluster, and the μ constant region. This 25 kb construct was randomly integrated into the mouse genome by microinjection into fertilized eggs. Approximately 4% of B cells expressed human μ chains, and hybridomas producing human IgM could be generated from these mice. Later, Taylor et al. introduced a human κ light chain construct containing a single Vκ, Jκ cluster, and Cκ [66]. Mice co-expressing the human VH-D-JH-Cμ-Cγ1 and κ constructs were able to produce human antibodies, though at levels below 10% of total Ig, reflecting limited compatibility with endogenous murine Ig expression [66]. In parallel, knockout mouse models were developed to eliminate endogenous murine Ig production. In 1993, Chen et al. disrupted the murine JH and Jκ loci by targeted deletion, abolishing native Ig expression [67,68]. When crossed with human IgH and IgL transgenic lines, these knockout mice displayed broader human antibody repertoires. A major advance came in 1994 when Lonberg et al. generated HuMabMouse, the first mouse line carrying human IgH and Igκ genes in a murine Ig-deficient background. Although the full human IgH and Igκ loci span 1.29 Mb and 1.39 Mb, the introduced constructs were less than 80 kb, which constrained antibody diversity. Subsequently, yeast artificial chromosome (YAC) technology was applied. In 1993, researchers successfully assembled large genomic fragments of human Igκ (~300 kb) and IgH (~85 kb) using YACs [69,70]. Subsequently, Green et al. introduced YAC-based human Igκ (~170 kb) and IgH (~220 kb) into mouse ES cells via yeast spheroplast–ES fusion. Building on this, Mendez et al. generated even larger YAC constructs, including human Igκ (~700 kb) and IgH (~1 Mb), and introduced them into Ig-deficient mice, creating XenoMouse [71]. These mice expressed only fully human antibodies, free from murine Ig interference. Together, the development of transgenic animals provided groundbreaking platforms that enabled the efficient generation of fully human therapeutic antibodies. Beyond XenoMouse, an increasing number of advanced transgenic mouse platforms have been developed, such as the Atlas™ Mouse, the HuMab Mouse, and the VelocImmune^®^ Mouse (Regeneron, Tarrytown, NY, USA). These models employ precise gene knock-in strategies to replace murine heavy- and light-chain variable regions with near-comprehensive human sequences, thereby enhancing antibody diversity while preserving natural structural characteristics. Newer generations also incorporate innovations such as fixed light-chain or binary light-chain strategies, which streamline the efficient generation of bispecific antibodies and improve developability and pharmacokinetic properties. Collectively, these innovations expand the functional versatility of therapeutic antibodies and provide greater flexibility in drug development pipelines. Although the technology remains costly and technically demanding, such transgenic mouse models are rapidly becoming an industry standard, demonstrating substantial value in addressing complex diseases and meeting high clinical demands [71,72,73,74].

## 5. Single B Cell Technology

In the human immune system, antibody responses are robust, highly specific, and often potently neutralizing. Traditional strategies to generate therapeutic monoclonal antibodies, such as murine hybridoma technology or the use of transgenic mice, require lengthy immunization protocols and extensive screening. Moreover, murine-derived antibodies carry a significant risk of immunogenicity in humans, frequently leading to the development of human anti-mouse antibody (HAMA) responses. To overcome these limitations, an early alternative was the immortalization of human B cells using Epstein–Barr virus (EBV) transformation [75]. While this approach enabled the production of human antibodies under certain conditions, it suffered from key drawbacks, including inefficiency in some donors and instability of EBV-transformed clones. A transformative advance came with single B cell technologies, which allow direct recovery of human antibodies without animal immunization. Using reverse transcription polymerase chain reaction (RT-PCR), the variable heavy (VH) and light (VL) chains from individual B cells can be amplified and expressed recombinantly [76,77]. B cells are typically isolated from peripheral blood mononuclear cells (PBMCs), bone marrow, or lymphoid tissues, often after density gradient centrifugation. Antigen-specific B cells are identified and sorted using FACS based on stage-specific markers. Earlier methods employed antigen-coated beads to enrich for rare B cells, leading to the first human monoclonal antibodies against viral pathogens [78]. Today, multiparameter flow cytometry and high-throughput single-cell cloning enable rapid identification and expression of paired VH/VL genes. These methods have revolutionized antibody discovery by offering an efficient, animal-free strategy particularly suited to urgent public health crises like emerging viral outbreaks such as pandemic influenza [7,8,79]. Applications of this strategy have been widely demonstrated. For example, broadly neutralizing antibodies (bnAbs) against HIV-1 were isolated from infected or vaccinated individuals by using HIV envelope proteins as baits [80,81,82,83]. These antibodies often target conserved epitopes on viral membrane glycoproteins, enabling potent and cross-reactive neutralization. More recently, single B cell methods have enabled the rapid identification of potent neutralizing antibodies against SARS-CoV-2. Panels of antibodies targeting the spike protein were isolated and characterized shortly after the COVID-19 pandemic began, providing critical insights into both therapeutic and vaccine design [84,85,86]. Collectively, single B cell antibody discovery platforms represent a transformative advance compared to traditional hybridoma methods, enabling faster, more direct, and clinically relevant recovery of fully human monoclonal antibodies.

## 6. De Novo Synthesis

Traditional antibody discovery and optimization techniques, such as hybridoma technology, phage display, transgenic mice, B-cell cloning, etc., are widely used for therapeutic antibody screening. However, some of these are labor-intensive, time-consuming, and costly, it usually takes more than six months to produce feasible antibodies [87,88]. Subsequently, through homology modeling, molecular docking, and structure-based design strategies, optimization steps were performed to enhance binding affinity, biophysical stability and developability [89,90,91,92]. Recent advances in protein data acquisition, GPU computers, and machine learning (ML) are expected to revolutionize the process of antibody discovery and optimized screening. The availability of extensive protein structure, interaction, and function data provides a large dataset for training sophisticated machine learning models, while enhanced computational power enables efficient execution of complex models and simulations. Machine learning models have been widely used in protein research, covering technologies such as neural networks, transducers, and protein language modeling [93,94]. De novo antibody design refers to the generation of new antibody sequences without relying on natural templates. Recent studies have used this approach to computationally predict sequences with high binding affinity based on accurate modeling of intermolecular interactions by simulating the antigen-antibody interaction interface [95]. The AlphaFold3 [13,96] and RoseTTAFold [97,98] models have achieved high accuracy in predicting protein structures directly from amino acid sequences. Generative models such as ProteinBERT [99], ProteinMPNN, [100,101] and RFdiffusion [102] have advanced computational protein designs by predicting protein backbones, designing sequences for specific structures, and filtering low-quality protein candidates. Researchers can use these models to design and optimize proteins with desired properties, such as enzymatic activity and binding. Specialized models focus on antibody design, particularly targeting the complementarity-determining regions (CDRs). IgFold can rapidly predict antibody structures using pre-trained language models and graph neural networks [103], and models like DiffAb can be used for the joint generation of antibody sequences and structures targeting CDR optimization [104].

**Figure 3:**
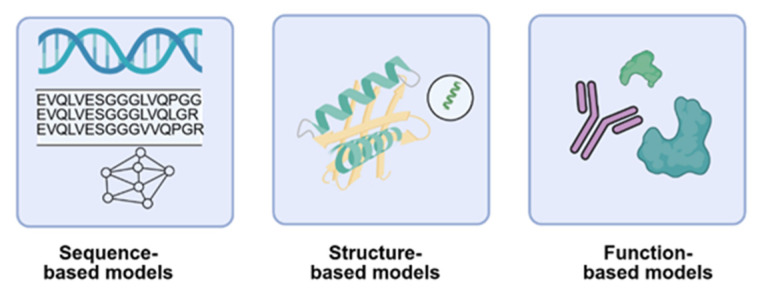
Schematic of machine learning modalities for antibody design. Sequence-based models leverage amino acid sequences to learn representations and generate novel variants and enabling de novo design. Structure-based models incorporate structural constraints to guide sequence generation and optimization. Function-based models utilize experimental, published data or predicted functional protein such as catalytic activity or binding for supervised learning.

Machine learning for functional protein design spans three core modalities: sequence-based, structure-based, and function-based models (Figure 3).

**Figure 4:**
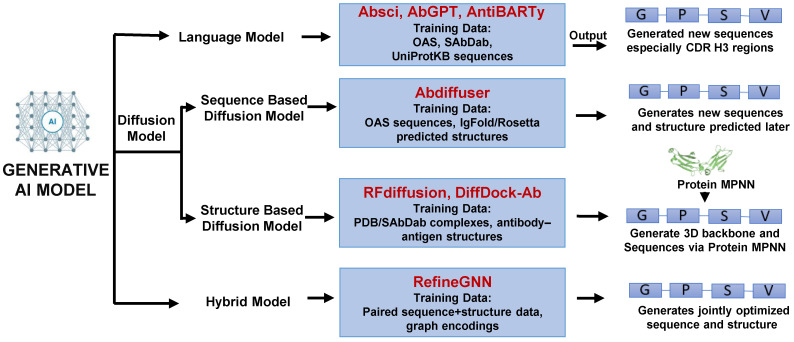
Schematic diagram of antibody generation using generative AI models. Generative AI approaches include Language models (sequence-based design), Diffusion models (sequence- or structure-guided generation), and Hybrid models (integrating sequence and structure for simultaneous optimization). An integrative framework transforms antibody discovery from published empirical screening to data-driven design.

Each leveraging distinct data type to optimize protein properties. Sequence-based models include classical alignment-based approaches using multiple sequence alignments to capture evolutionary constraints, and conditional generative models like variational autoencoders and transformers that generate novel sequences based on family-specific or sequence homology contexts [105,106]. A Generative Adversarial Network (GAN) was designed [107] and trained on over 400,000 human antibody light and heavy chain sequences effectively learns the underlying principles of antibody formation [108]. Structure-based models encompass structure prediction tools such as AlphaFold3 and RoseTTAFold [97], RF-diffusion models [109] for 3D fold creation, and design models that optimize sequences within structural frameworks. David Baker’s team recently developed RFdiffusion, a generative model for de novo design of VHHs and scFvs targeting influenza hemagglutinin and Clostridium difficile toxin B (TcdB). It achieves atomic-level precision in both structure and epitope targeting. [110]. These approaches integrate sequence and structural data are increasingly used to iteratively refine antibody designs, combining the strengths of both modalities for enhanced functional outcomes [111]. Function-based models focus on engineering antibodies with specific biological activities, often starting from known scaffolds and introducing targeted mutations to enhance binding, specificity. These approaches frequently reference receptor pockets, natural ligands, or agonist, antagonist interactions to guide the design of functional antibodies [112]. These models enable rational design of agonists, antagonists, and therapeutic proteins. Together, these modalities offer complementary strategies for accelerating protein engineering, with growing applications in antibody design, optimization, and synthetic biology. Collectively, recent strategies converge within generative AI frameworks that can be broadly classified into three categories: Language Models, Diffusion Models, and Hybrid Models (Figure 4).

Language models (e.g., Absci, AbGPT, AntiBARTy) [113,114] are trained on large antibody sequence repositories to generate novel sequences, particularly within highly diverse regions such as CDRH3. Diffusion models are subdivided into structure-based (e.g., RFdiffusion, DiffDock-A) [110,115] which use experimentally solved antibody–antigen complexes from PDB or SAbDab to design new 3D backbones followed by sequence optimization via Protein MPNN, and sequence-based (e.g., Abdiffuser) [116] which leverage antibody sequence datasets with predicted structures to generate novel sequences. Hybrid models (e.g., Refine GNN) [117] integrate paired sequence and structure data, enabling the simultaneous optimization of both sequences and structures. Together, these generative frameworks provide complementary strategies that expand the landscape of antibody discovery and accelerate the design of functional therapeutic candidates.

Now, AI-assisted antibody design has demonstrated remarkable versatility across diverse targets, including virus proteins, membrane receptor oncogenic proteins [117,118,119,120]. As computational modeling and high-throughput experimentation converge, de novo antibody generation is poised to become an automated, iterative process driven by machine learning and structural bioinformatics. This integration promises faster, more precise development of therapeutic antibodies, accelerating breakthroughs in cancer, infectious diseases, and immunotherapy. AI-assisted antibody design represents a new trend that is reshaping antibody discovery [110,121].

## 7. Summary and Outlook

This review highlights diverse strategies for antibody discovery, including hybridoma technology, phage display libraries, transgenic mice, single B cell isolation, and de novo synthetic approaches. Despite these advances, each antibody discovery approach still presents unique challenges and trade-offs. Hybridoma methods are limited by species-specific immune responses such immune tolerance, while phage display may introduce library bias and lose native antibody pairing. Transgenic mouse platforms are costly, and single B-cell technologies face throughput and data interpretation constraints. Moreover, current AI-driven and de novo computational design approaches still struggle with accurately predicting antibody folding, dynamics, and functional efficacy, underscoring the need for integrated, multi-platform strategies in next-generation antibody development. In addition, beyond these five antibody generation strategies, post-production engineering such as Fc modification, half-life extension, and glycoengineering also continues to enhance antibody efficacy, stability, and therapeutic index. These innovations complement discovery platforms, bridging antibody generation with clinical optimization for next-generation biologics. Taken together, in this review, we summarize five major antibody generation methodologies that are expanding the frontiers of therapeutic antibody discovery, providing innovative strategies to accelerate the development process and enhance the therapeutic efficacy of human diseases. Collectively, these platforms have enabled the rapid generation of not only conventional neutralizing antibodies but also functional agonists that modulate signaling pathways and drive cellular differentiation. Emerging methods such as autocrine-based and migration-based selection, in vivo functional screening, and morphology-guided assays further expand the potential of antibody discovery, particularly in cancer and immunology. These advances provide powerful means to target viruses, tumors, and immune receptors, while also offering new insights into receptor pleiotropy and cell fate regulation. De novo design, increasingly coupled with AI-driven modeling and high-throughput screening, represents a new trend that is reshaping antibody discovery. Looking forward, the convergence of these approaches with next-generation computational tools will accelerate the development of innovative therapeutic antibodies across infectious diseases, oncology, and beyond.

## Data Availability

No new data were created or analyzed in this study. Data sharing is not applicable to this article.
